# A polymorphism in the insulin-like growth factor 1 gene is associated with postpartum resumption of ovarian cyclicity in Holstein-Friesian cows under grazing conditions

**DOI:** 10.1186/1751-0147-55-11

**Published:** 2013-02-14

**Authors:** Paula Nicolini, Mariana Carriquiry, Ana Meikle

**Affiliations:** 1 Laboratory of Nuclear Techniques, Faculty of Veterinary Medicine, University of Uruguay, Lasplaces 1550, C.P, Montevideo 11600, Uruguay; 2 Faculty of Agronomy Sciences, University of Uruguay, Av. Garzón 780, C.P, Montevideo 12900, Uruguay

**Keywords:** IGF-1, Polymorphism, Commencement of luteal activity, Cattle

## Abstract

**Background:**

Insulin-like growth factor 1 (*IGF-1*) gene is considered as a promising candidate for the identification of polymorphisms affecting cattle performance. The objectives of the current study were to determine the association of the single nucleotide polymorphism (SNP) *IGF-1/Sna*BI with fertility, milk production and body condition traits in Holstein-Friesian dairy cows under grazing conditions.

**Methods:**

Seventy multiparous cows from a commercial herd were genotyped for the SNP *IGF-1/Sna*BI. Fertility measures evaluated were: interval to commencement of luteal activity (CLA), calving to first service (CFS) and calving to conception (CC) intervals. Milk production and body condition score were also evaluated. The study period extended from 3 wk before calving to the fourth month of lactation.

**Results and discussion:**

Frequencies of the SNP *IGF-1/Sna*BI alleles A and B were 0.59 and 0.41, respectively. Genotype frequencies were 0.31, 0.54 and 0.14 for AA, AB and BB, respectively. Cows with the AA genotype presented an early CLA and were more likely to resume ovarian cyclicity in the early postpartum than AB and BB ones. No effect of the SNP *IGF-1*/*Sna*BI genotype was evidenced on body condition change over the experimental period, suggesting that energy balance is not responsible for the outcome of postpartum ovarian resumption in this study. Traditional fertility measures were not affected by the SNP *IGF-1*/*Sna*BI.

**Conclusion:**

To our knowledge this is the first report describing an association of the SNP *IGF-1*/*Sna*BI with an endocrine fertility measure like CLA in cattle. Results herein remark the important role of the *IGF-1*gene in the fertility of dairy cows on early lactation and make the SNP *IGF-1*/*Sna*BI an interesting candidate marker for genetic improvement of fertility in dairy cattle.

## Background

Over the last decades fertility in Holstein dairy cows has declined in coincidence with the continuing drive by breeding companies to increase genetic merit for milk production [1,2]. The concern of poor fertility is such that in recent years many countries have implemented genetic evaluations for fertility traits [3-5]. Traditional fertility measures, such as calving-to-first service (CFS) and calving-to-conception (CC) intervals, are difficult to use in genetic improvement programmes because: 1) fertility is expressed late in life, 2) reproductive records are lacking and/or have poor quality, 3) traits are not normally distributed [6], which complicates the analysis, and 4) have low heritability (<5%) [4]. Moreover, traditional reproductive traits are highly influenced by management e.g., CFS - an indirect measure of the interval from calving to first ovulation - is affected by farmer’s decisions on the timing of inseminations, which vary between herds and even between cows within the same herd [7].

The interval from calving to commencement of luteal activity postpartum (CLA), determined by milk progesterone (MP_4_) profiles, has been suggested as a suitable selection criteria for fertility, as early CLA is an important factor for a new pregnancy after calving, CLA is free from management bias, it presents higher heritability values (16 to 25%) than traditional fertility traits, and it is genetically favorably correlated with traditional fertility traits [8-11].

The delay in the resumption of ovarian cyclicity postpartum has been associated with a low reproductive performance in both confinement [12] and grazing systems [13]. The energy balance has a major effect on the resumption of cyclicity in the postpartum dairy cow [14]. The metabolic hormone Insulin-like growth factor 1 (IGF-1) is believed to be one of the main mediators of the effects of energy balance on the reproductive performance of the dairy cow after calving [15], since circulating concentrations of IGF-1 are highly associated to energy balance [16,17], follicular growth [18,19] and resumption of ovarian cyclicity [13,20-22].

Therefore, *IGF-1* is considered as a promising candidate gene for the identification of molecular markers predicting reproductive performance in cattle. Nevertheless, there is a scarcity of information available on polymorphisms in bovine *IGF-1* gene and their effects on economically important traits in beef and dairy cattle. The most studied *IGF-1* gene variation is the single nucleotide polymorphism (SNP) *IGF-1*/*Sna*BI, in the promoter region of the gene (512-bp 5^′^ to the first codon of the first exon, GeneBank Accession No. AF017143) [23]. In dairy cattle, some studies have analyzed the association of this SNP with milk production traits [24-29]. However, studies evaluating the effect of this SNP on fertility traits in dairy cows are even more recent and scarce [28-30], with only the work from Ruprechter et al. [29] reporting a significant effect of the SNP *IGF-1*/*Sna*BI on CFS interval in primiparous Holstein-Friesian (HF) cows. To our knowledge there is no other published information showing a significant effect of this SNP on reproductive performance either in beef or dairy cattle. Based on these considerations, we examined the effects of the SNP *IGF-1/Sna*BI on CLA and traditional fertility measures, as well as on body condition score and production traits in multiparous HF dairy cows under a seasonally calving pasture-based system.

## Materials and methods

### Animals

All procedures were carried out in accordance with regulations set by the “Comisión Honoraria de Experimentación Animal (CHEA)”, University of Uruguay, Uruguay. Seventy HF cows (n = 42 Uruguayan HF (UHF), and n = 28 UHF x New Zealand HF (NZHF) first cross (UHFxNZHF), i.e., progeny of NZHF sires) in their second (L2, n = 46) and third (L3, n = 24) parity, that calved with normal parturitions between late May and middle August, and that were free of detectable reproductive disorders and of clinical disease during the experimental period, were selected from a commercial farm in Uruguay. Average herd production was about 6800 L/cow/year.

### Diet

Before and after calving, cows grazed as one herd on improved pastures (mixture of grasses and legumes) for 6 h/day. In addition, during ap cows were fed *ad libitum* a diet that consisted in whole-plant grain sorghum silage (33% dry matter, DM; 1.1 MCal net energy for lactation, NEL/kg DM, 6.8% crude protein, CP), high moisture sorghum grain (16% CP, 1.5 MCal NEL/kg DM) and a commercial mineral supplement. After calving, cows were maintained on the same ap diet and supplemented with 7 (June), 15 (July), 12 (August), 4 (September/October/November) and 6 (December) kg (DM) of a commercial concentrate (16% CP, 1.5 MCal NEL/kg) which was provided once a day after the morning milking.

### Reproductive management

A planned start of mating was imposed after a voluntary waiting period of 90 days from the beginning of the calving period. The breeding season extended for three months (September to November) in order to achieve a concentrated calving pattern in the following season. Artificial insemination was performed for the first two months followed by a further month of natural mating. Cows were inseminated 12 h after heat detection, Pregnancy diagnosis was performed by palpation per rectum 45 days after insemination.

### Milk and body condition score measurements

Body condition score was performed by two trained persons according to Edmonson et al. [31] using a 5-point scale (1 = emaciated, 5 = fat) with quarter-point increments. Cows were scored fortnightly from 3 wk before the expected calving date to the fourth month of lactation. The final score per cow at each time-point observation corresponded to the average value of the scores given by each scorer. Cows were milked twice daily, and milk yield and composition were determined from morning and afernoon herd-tests samples once monthly until the fourth month of lactation.

### Milk sampling for MP_4_ determination

Milk samples for P_4_ determination were collected twice weekly during the morning milking. For each cow, sampling began at calving (Day 0) and extended until cyclicity was detected. In the case of one cow no detectable MP_4_ was observed over the study period, thus, the last day of sampling (around Day 120) was arbitrarily considered as the initiation of ovarian cyclicity for that cow, as previously defined by Meikle et al. [13]. Milk samples (20 mL) were collected into plastic tubes containing NaN_3_ as a preservative, refrigerated, transported to the Laboratory of Nuclear Techniques (Veterinary Faculty, Uruguay), and held at 4°C up to one week. Samples were skimmed by centrifugation at 3000 rpm at 4°C for 15 min and skim milk samples were stored at −20°C until P_4_ analysis. Progesterone was determined by a solid radioimmunoassay using a commercial kit (Coat-a-count, DPC, Diagnostic Products Co., Siemens, Los Angeles, CA, USA). The intra and inter-assay CVs of a medium control (4.5 nmol/L) were 5.8% and 9%, respectively. The sensitivity was 0.15 nmol/L.

### Reproductive measurements

The endocrine fertility measure CLA interval (days, n = 70) was defined as the number of days from calving to the first day of luteal activity (i.e., the date of the first of two consecutive records where MP_4_ concentrations increased above the threshold value of 1 nmol/L). Traditional fertility measures such as CFS (days, n = 55) and CC (days, n = 42) intervals were obtained from herd’s records on actual calving and service dates, and on pregnancy diagnosis data, respectively. Culling of cows due to reproductive failure reduced the original sample size to the numbers indicated in parentheses for CFS and CC measures.

### DNA extraction and SNP *IGF-1*/*Sna*BI genotyping

Blood samples (10 mL) were collected from HF cows by coccygeal venipuncture in K_2_EDTA Vacutainer® tubes (Becton Dickinson, NJ, USA) and stored at 4°C for 48 h until DNA isolation at the Laboratory of Nuclear Techniques, Veterinary Faculty, Uruguay. Genomic DNA was isolated from whole blood using a proteinase K/salting out/ethanol precipitation procedure as described by Montgomery and Sise [32]. Quantity and quality of DNA were assesed using a NanoDrop™ ND-1000 UV–vis spectrophotometer (NanoDrop Technologies, Inc., Wilmington, DE). DNA samples were stored at −20°C until assayed. Cows were genotyped for the SNP *IGF-1/Sna*BI by the amplification of a 249-bp fragment with the primers: forward 5^′^-ATTACAAAGCTGCCTGCCCC-3^′^ and reverse 5^′^-ACCTTACCCGTATGAAAGGAATATACGT-3^′^, followed by digestion with *Sna*BI restriction endonuclease (New England Biolabs, USA), accordig to Ge et al. [23]. Analysis of the restriction fragments of A (nucleotide *T*) (223 and 26 bp) and B (nucleotide *C*) (undigested, 249 bp) alleles was performed by 3% agarose gel electrophoresis at 100 V for 1 h, followed by ethidium bromide staining and UV visualization.

### Statistical analyses

Allele and genotype frequencies of the SNP *IGF-1/Sna*BI, as well as Hardy-Weinberg equilibrium (HWE) test and Ewens-Watterson test for neutrality were performed for each genetic strain (UHF, UHFxNZHF) and for the total sample, using the PopGene32 v1.31 program [33]. Differences in the distribution of allele and genotype frequencies between genetic strains were also estimated using the same program. All other variables were analyzed using SAS programs (SAS 2000, SAS Institute Inc., Cary, NC, USA). Univariate analyses were performed on all variables to identify outliers and inconsistencies and to verify normality of residuals. Homogeneity of variances was verified with Levene’s test. Changes in body condition and milk, 4% fat-corrected milk and total solids yields (MY, FCM and TSY, respectively), were analyzed as a complete randomized design by repeated measures using the mixed procedure (PROC MIXED) with a first order autoregressive covariance structure specified. Days categorized in 20-day intervals before and after calving (Day 0), and in 31-day intervals from calving, were used as the repeated effect, respectively. The model included the fixed effects of SNP *IGF-1*/*Sna*BI genotype (AA, AB, BB), strain (UHF, UHFxNZHF), parity (L2, L3), day interval, and interactions. Calving date and body condition at calving were used as covariates. Sires were included as random effect. Fertiliy measures (CLA, CFS and CC intervals) were analyzed using a generalized linear model (PROC GENMOD) with a Poisson distribution and log transformation specified in a model that included the fixed effects of the SNP *IGF-1*/*Sna*BI genotype, strain, parity, and interactions. Calving date, average MY over the study period and body condition at calving were used as covariates. The Kenward-Rogers procedure was used to adjust the denominator degree of freedom to test significance level of fixed effects. Transformed estimates were back transformed for presentation. The probability of cows cycling at Day 30, 45, 60, 90 and 120 was analyzed using a generalized lineal model (PROC GENMOD) with a binomial distribution and an exchangeable covariance structure specified. Means were separated using the Tukey-Kramer procedure. Interactions were retained in the model if P < 0.1. The reduced model was used in the final analysis. All data from models are presented as least square means (LSM) ± pooled standard error. For those traits significantly affected by the SNP *IGF-1*/*Sna*BI genotype, additive effects were estimated by subtracting LSMs for BB genotype from those for AA genotype; whereas dominance effects were estimated by subtracting the average of LSMs for homozygous genotypes from those for AB genotype [24]. Average allele substitution effects (i.e., substitution of one allele in the population with the other allele) [34] were estimated using the same models used to estimate the genotype effects but replacing the classification effect of genotypes by a linear regression on the number of A alleles (BB = 0, AB = 1, or AA = 2). Significance was set at P ≤ 0.05, and a trend towards significance was set at P ≤ 0.1.

## Results

### SNP IGF-1/SnaBI allele and genotype frequencies

**Figure 1:**
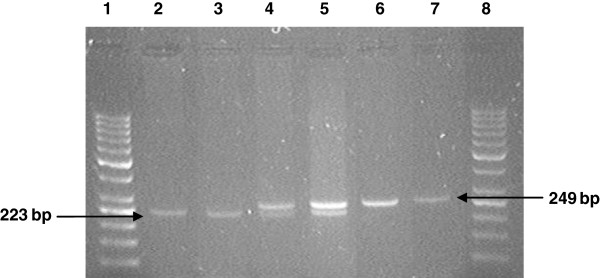
**Agarose gel electrophoresis showing the SNP *IGF-1*/*Sna*BI in 5**^**′**^**-noncoding region of cattle *IGF-1 *gene. **Lanes 1 and 8: molecular size marker 50-bp ladder (Fermentas); lanes 2 and 3: AA (*TT*) genotype (223-bp); lanes 4 and 5: AB (*TC*) genotype (249 and 223-bp); lanes 6 and 7: BB (*CC*) genotype (249-bp). The 26-bp fragment in AA and AB genotypes is undetectable.

Seventy HF cows from two genetic strains were genotyped for the SNP *IGF-1/Sna*BI at the 5^′^-promoter region of the *IGF-1* gene. Digestion of the 249-bp PCR product with *Sna*BI resulted in the detection of the two alleles (A and B) and the three possible genotypes (AA, AB and BB) for this SNP (Figure 1). Genotype AA (*TT*) was characterized by the presence of one restriction fragment of 223-bp, whereas genotype AB (*TC*) presented two bands of 249- and 223-bp. In both cases, the 26-bp band was undetectable in the electrophoresis. The PCR product from the homozygous genotype BB (*CC*) remained undigested at 249-bp. Allele and genotype frequencies of the SNP *IGF-1/Sna*BI calculated for each genetic strain, as well as for the total sample, are shown in Table 1. The A allele and the AB genotype were the most frequent in both genetic groups. Genotype frequencies tended to differ between these groups (P = 0.1), with UHF cows showing higher frequencies of the AB genotype and lower frequencies of the BB genotype than UHFxNZHF group (Table 1). Although frequency of the B allele was lower in the UHF group than in UHFxNZHF cows, differences did not reach significance (P = 0.18). The SNP *IGF-1/Sna*BI genotype frequencies conformed to HWE proportions for the UHFxNZHF strain and the total sample (Table 1). However, this locus tended to deviate from HWE in the UHF sample, which tended to exhibit more cows with the heterozygous genotype than expected (Table 1). Nevertheless, results from the Ewens-Waterson test did not assess departure from neutrality in this sample, relative to the 95% CI for expected homozygosity at the locus.

**Table 1:** **Allele and genotype frequencies observed for the SNP *IGF-1*/*Sna*BI in Holstein-Friesian cows**

**SNP *IGF-1/Sna*BI**	**UHF**^**1**^	**UHFxNZHF**^**2**^	**Total**^**3**^
allele frequencies			
A	0.63	0.52	0.59
B	0.37	0.48	0.41
genotype frequencies			
AA	0.33 (14)	0.28 (8)	0.31
AB	0.59 (25)	0.46 (13)	0.54
BB	0.07 (3)	0.25 (7)	0.14

*UHF*: Uruguayan Holstein-Friesian cows; *UHFxNZHF*: UHF x New Zealand Holstein-Friesian first cross cows. ^1,2, 3^ Hardy-Weinberg Equilibrium test: *χ*^2^ = 2.98, P = 0.08; *χ*^2^ = 0.22, P = 0.64; *χ*^2^ = 0.87, P = 0.35, respectively. The number of animals per genotype is indicated in parentheses.

### Body condition score and milk production

Neither body condition score nor milk production traits were affected by the SNP *IGF-1*/*Sna*BI genotype or strain. Body condition score declined (P < 0.0001) around calving (3.2 ± 0.03 points), reached its lowest value around Day 45 (2.8 ± 0.03 points), and started to recover at Day 95 (2.9 ± 0.03 points).

### Fertility measures

Significant genotype, additive and allele substitution effects of the SNP *IGF-1*/*Sna*BI were observed on interval to CLA, with homozygous AA genotype being more favorable for this trait (Table 2). Cows carrying the AA genotype resumed postpartum ovarian cyclicity on average ~ 15 and 33 days earlier than AB and BB ones, respectively; in addition, AB cows tended to exhibit shorter intervals than BB ones (Table 2). A trend for a dominance effect of the A allele was observed, as heterozygous cows presented shorter intervals to CLA than the average for homozygous cows (Table 2). No strain or parity effects were observed on CLA. No effect of either body condition at calving, average MY over the study period or calving date (covariates) were observed on CLA.

**Table 2:** **Least square means (± pooled standard error) of SNP *IGF-1*/*Sna*BI-associated effects on productive and reproductive traits in Holstein-Friesian cows**

	**SNP *IGF-1*/*Sna*BI genotype**			**Additive effect**	**P-value**	**Dominance effect**	**P- value**	**Allele substitution effect**	**P- value**
	**AA**	**AB**	**BB**						
***Production***^**1 **^**(n = 70)**									
Milk Yield (L/cow)	25.4 ± 0.7	26.3 ± 0.5	25.7 ± 1.1	−	−	−	−	−	−
4% Fat-Corrected Milk (L/cow)	22.8 ± 0.7	23.4 ± 0.5	23.8 ± 0.9	−	−	−	−	−	−
Total Solids Yield (kg/cow)	2.9 ± 0.1	2.9 ± 0.06	2.9 ± 0.1	−	−	−	−	−	−
***Fertility *(days)**									
Calving-CLA (n = 70)	35.0 ± 6.0^ax^	49.8 ± 4.5^bx^	67.8 ± 8.6^by^	−32.8 ± 10.5	0.003	−1.6 ± 9.8	0.07	−16.0 ± 5.0	0.002
Calving-First Service (n = 55)	83.2 ± 4.5	81.6 ± 3.3	79.0 ±6.7	−	−	−	−	−	−
Calving-Conception (n = 42)	90.8 ± 5.5	86.7 ± 3.7	92.0 ± 7.0	−	−	−	−	−	−

^1 ^Production traits represent the first four months of lactation. *CLA*: commencement of luteal activity. Means with different superscripts within the same row differ: a vs b P ≤ 0.04, x vs y P ≤ 0.09.

**Figure 2:**
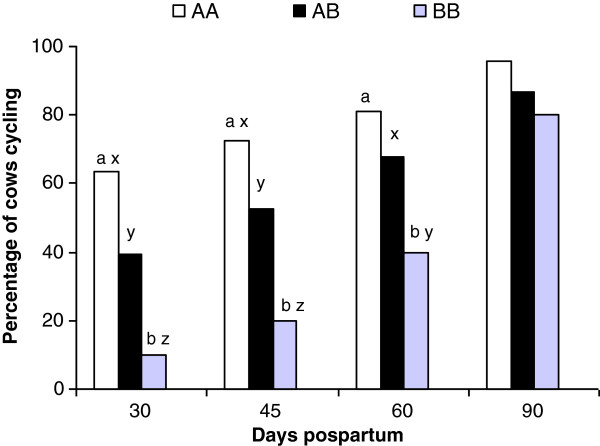
**Percentage of cows cycling across SNP *IGF-1*/*Sna*BI genotypes (AA, AB, BB) within the same day postpartum. **Superscripts differ by: ^a, b ^P ≤ 0.05, ^x, y, z^ P ≤ 0.1.

Probability of cows cycling was affected by the SNP *IGF-1*/*Sna*BI genotype on Day 30 (P = 0.01), 45 (P = 0.02) and 60 (P = 0.06), but not on Day 90 (Figure 2). Cows with AA genotype were more likely to resume ovarian cyclicity in the early postpartum period (before Day 45) than those with AB or BB genotypes. Differences between AA and BB cows were significant on Day 30, 45 and 60. The AA genotype tended to present more cows cycling than AB genotype on Day 30 (P = 0.07) and 45 (P = 0.1), whereas a trend was observed for the AB genotype showing more cows cycling than the BB genotype on Day 30 (P = 0.1), 45 (P = 0.08) and 60 (P = 0.1).

Neither CFS nor CC interval was affected by the SNP *IGF-1*/*Sna*BI genotype, strain or parity (Table 2). Average milk yield over the study period (covariate) did not affect CFS, but tended to affect CC interval (P = 0.09), as cows with shorter intervals tended to have higher production.

## Discussion

The SNP *IGF-1*/*Sna*BI has been studied in about 30 cattle breeds, including dairy and beef breeds [23,24,27,28,35-38]. In the current study, allele frequencies of the SNP *IGF-1*/*Sna*BI estimated for the UHFxNZHF group (A: 0.52, B: 0.48) as well as for the total sample (A: 0.59, B: 0.41), are in agreement with those previously published in North American (NA) [24], Polish [25], and Uruguayan [29] HF populations (A: 0.55, 0.52 and 0.60; B: 0.45, 0.48 and 0.40, respectively). On the other hand, allele frequencies in the sample of UHF cows tended to present a different distribution (A: 0.63, B: 0.37), which is similar to that (A: 0.64, B: 0.36) reported by Ge et al. [23] in Angus beef cattle. In our study, the sample of UHF cows tends to deviate from HWE due to a heterozygote excess. Reasons for this tendency could be genetic drift or the action of selection acting on this sample; however, no action of selection on the *IGF-1* locus was evidenced in our study. Likewise, deviations from HWE were reported for the *IGF-1* locus in both beef and dairy cattle [39,40] and, in particular, for the SNP *IGF-1*/*Sna*BI in HF populations [27,36]. Furthermore, evidence of selection acting on the *IGF-1* locus were found in Swedish and Polish HF populations [36], as well as in the brazilian Canchim beef cattle breed [39]. However, evidence of selection acting on this locus might be even greater provided it has not been investigated in most published works on cattle *IGF-1* polymorphisms.

Our results from association analyses of the SNP *IGF-1*/*Sna*BI with milk production traits show no significant effects of this SNP on either MY, FCM or TSY within the first four months of lactation. This is in accordance with previous reports that failed to observe any significant association between the SNP *IGF-1*/*Sna*BI and dairy production traits in HF cattle [24,28]. On the other hand, results from Mehmannavaz et al. [27] proved a significant effect of the SNP *IGF-1*/*Sna*BI on estimated breeding values (EBV) for milk production traits in Iranian Holstein bulls, as animals with AB genotype had higher EBV for milk and fat yields than homozygous genotypes. In addition, Siadkowska et al. [25] and Bonakdar et al. [26] reported associations between the AB genotype and higher percentage of milk fat and protein in Polish and Iranian HF cows, respectively. Likewise, previous studies from our group have shown that AB cows tended to yield more FCM than primiparous BB cows at early and middle lactation and than multiparous AA cows over the lactation period [29]. No other reports were found in the literature concerning the effect of the SNP *IGF-1*/*Sna*BI on milk production traits.

The results of the present study further indicate a significant association of the SNP *IGF-1*/*Sna*BI with interval to CLA. Cows with the AA genotype present a shorter interval to CLA and are more likely to resume ovarian cyclicity in the early postpartum than AB and BB ones. Potential mechanisms for an association between the SNP *IGF-1*/*Sna*BI and the traits of interest may lie in its location in the promoter region of the *IGF-1* gene and its possible influence on transcription factor binding sites and on gene expression [28,41,42]. Higher IGF-1 blood levels have been found in cows exhibiting shorter CLA intervals [13,43]. Association studies between the SNP *IGF-1*/*Sna*BI and plasma IGF-1 concentration in cattle are contradictory. Ge et al. [23] reported lower IGF-1 levels in young BB Angus bulls, while Maj et al. [41] and Mirzaei et al. [42] found higher IGF-1 concentrations in BB Polish and Iranian HF cattle, and Ruprechter et al. [29] reported no association in Uruguayan HF cows. Inconsistences among studies may be explained by IGF-1 having different effects according to breeds, stages of growth, physiological states and nutritional status, as it is known that *IGF-1* gene expression is developmentally and physiologically regulated [44].

Circulating IGF-1 concentrations are temporarily affected by the negative energy balance, which in dairy cows dramatically changes during the transition period. Meikle et al. [13] reported that, under grazing conditions, lean cows with reduced serum IGF-1 concentrations presented prolonged postpartum anestrus, showing that circulating IGF-1 levels are good indicators of the re-initiation capacity after calving, which is in accordance with others [45,46]. In our study, although AA and AB cows presented shorter anestrus periods than BB ones, no differences were observed in body condition at calving among them. Moreover, differences in body condition change during the experimental period were not evidenced among genotypes, which suggests that energy balance measured by changes in body condition do not explain the differences observed among the SNP *IGF-1*/*Sna*BI genotypes on CLA interval after calving. In addition, the lack of a significant effect of milk yield on CLA could be consider as a further evidence that the energy balance is not responsible for the outcome of postpartum ovarian resumption in this study.

Although our results showed an effect of the SNP *IGF-1*/*Sna*BI on CLA, no effect was observed on traditional fertility measures like CFS and CC intervals. This is in agreement with the fact that CLA reflects more directly the reproductive physiology of the cow, being more influenced by the cow itself than traditional fertility measures [47], which are highly influenced by management and farmer’s decisions [7]. Nevertheless, sample sizes analyzed in this study for traditional traits are insuficient to get to concluding results. To our knowledge this is the first report describing an association of the SNP *IGF-1*/*Sna*BI with an endocrine fertility measure like CLA in cattle. These results are consistent with findings from a previous work of our group in another HF herd [29], where longer CFS intervals were found in primiparous BB cows. In contrast, other studies have failed to identify any association of the SNP *IGF-1*/*Sna*BI with endocrine or traditional fertility traits in cattle [28,30]. No other reports were found in the literature concerning the effects of this SNP or other SNPs at the *IGF-1* gene on fertility traits in cattle.

## Conclusions

Our results indicate a significant association of the SNP *IGF-1*/*Sna*BI with interval to CLA in HF dairy cows in a pasture-based system. Results herein remark the important role of the *IGF-1*gene in the fertility of dairy cows on early lactation and make the SNP *IGF-1*/*Sna*BI an interesting candidate marker for genetic improvement of fertility in dairy cattle.

## Abbreviations

CC: Calving to conception interval; CFS: Calving to first service interval; CLA: Interval to commencement of luteal activity; CP: Crude protein; DM: Dry matter; EBV: Estimated breeding values; FCM: Fat corrected milk; HWE: Hardy-Weinberg equilibrium; IGF-1: Insulin-like growth factor 1; L2: Second parity; L3: Third parity; LSM: Least square means; MP_4_: Milk progesterone; MY: Milk yield; NEL: Net energy for lactation; NZHF: New Zealand Holstein-Friesian; SNP: Single nucleotide polymorphism; TSY: Total solids yield; UHF: Uruguayan Holstein-Friesian; UHFxNZHF: UHF x NZHF first cross.

## Competing interests

The authors declare that they have no competing interests.

## Authors’ contributions

PN contributed with the experimental design, carried out the hormone determinations and genotyping, and drafted the manuscript. MC contributed with the statistical analysis and manuscript corrections. AM lead the experimental design, contributed with data interpretation, statistical analysis and manuscript corrections. All authors read and approved the final manuscript.
